# Electrochemical Aptasensor Developed Using Two-Electrode Setup and Three-Electrode Setup: Comprising Their Current Range in Context of Dengue Virus Determination

**DOI:** 10.3390/bios13010001

**Published:** 2022-12-20

**Authors:** Mohd. Rahil Hasan, Pradakshina Sharma, Shifa Shaikh, Saumitra Singh, Roberto Pilloton, Jagriti Narang

**Affiliations:** 1 Department of Biotechnology, School of Chemical and Life Sciences, Jamia Hamdard, New Delhi 110062, India; 2 Institute of Crystallography of National Research Council (IC-CNR), 00118 Rome, Italy

**Keywords:** paper electrode setups, current amplification, dengue detection, comparative study

## Abstract

We present, for the very first time, the fabrication and electrochemical characterization of a paper-based experimental platform for dengue virus analysis. The paper-based device incorporates a screen-printing technology with the help of black carbon conductive ink. The paper-based device utilizes two styles of electrode setups, i.e., the two-electrode system and three-electrode system, and both setups effectively detected the dengue virus with an LOD of 0.1 µg/mL; however, these paper electrodes exhibit various current ranges, and the created sensor was encompassed and compared in this research based on current response. It is observed that the three-electrode system has a substantially higher current range, ranging from 55.53 µA to 322.21 µA, as compared to the two-electrode system, which has a current range of 0.85 µA to 4.54 µA. According to this study, the three-electrode system displayed a good range of current amplification that is roughly 50 times higher than the two-electrode system, which had a weak current response. As a result, the three-electrode method has emerged as a viable option for the very sensitive detection of the dengue virus, as well as for the diagnosis of other diseases.

## 1. Introduction

Electrochemical sensing for paper-based analytical devices (ePADs) is appealing because it can achieve low detection limits with good sensitivity while using compact, affordable instruments. Many electrode materials, including gold, silver, and carbon, have been employed in paper-based systems to date [1]. Among these, only carbon is an appealing material for electrode fabrication because of its inexpensive cost, ease of production, broad potential window, and inert nature, among others [2]. Carbon and carbon-based materials have a variety of qualities, including high elasticity, high thermal conductivity, and low density, which is why they play an important part in nanotechnology, electronics, optics, and other branches of materials research due to their intriguing features [3,4].

There are numerous types of diagnostics methods available for the detection of various diseases, such as PCR, ELISA, and many more, but these approaches are very expensive, time consuming, and less specific and need expertise. Therefore, to overcome these limitations, a newly emerging technique, i.e., biosensor, is required for the detection of target diseases as it is easy to use, good for on-site detection, rapid, and highly specific. Biosensors come in large varieties, such as electrochemical biosensors, lateral flow biosensors, colorimetric biosensors, etc. The electrochemical biosensor, which is one of the popular biosensors due to its accuracy, sharp reduction in sample volume, portability, and cost effectiveness, was obtained by miniaturizing solid electrodes. It was required to abandon the frequently employed bulky electrodes and cells to meet the demands of on-site analysis [5,6,7]. For electrochemical biosensors, electrodes are one of the main components as these are the conductors that are utilized to make electrical contact with a non-metallic component of the circuit. The name electrodes was initially used by William Whewell and is derived from two Greek words: hodos, which means “a way”, and elektron, which means “amber” [8]. Electrodes play a significant role in the field of diagnostics, and there are various electrode setups introduced by researchers, such as the bulky electrode setup, gold electrode-based setup, graphite electrode-based setup, and many more, but these setups of electrodes are very costly and are not suitable for the construction of low-cost biosensors [9]. Currently, many scientists are trying to develop low-cost biosensors by using cheap substrates such as paper [10]. In electrochemical biosensors, the price of biosensors can be reduced by including paper-based electrodes. Paper-based electrodes have numerous advantages over commercialized screen-printed electrodes available in the market as these are easy to fabricate, cheap, disposable, and easy to handle [11,12]. Sensors which have mainly two forms of electrode setups are used in the diagnostic industry; one is the two-electrode system, and the other one is the three-electrode system [13,14]. Numerous reports are available on both systems utilized by researchers such as santhiago et al. [1] who designed a two-electrode system-based electrochemical device for the detection of p-nitrophenol with a range of 10–200 µM. Other than two electrodes, three electrodes were significantly utilized in the determination of various targets. For example, Mehto et.al. [15] developed a three-electrode system for the detection of yellow fever with an LOD of 0.01 µM. Therefore, these reports proved that both systems of electrodes are significantly applicable for the detection of various targets.

The main components of the electrode system in a three-electrode system consist of three types of electrodes: (a) working electrode, (b) counter electrode, and (c) reference electrode [16]. The working principle and components of the two-electrode system are similar to those of the three-electrode system. The sole difference between these two configurations is that the two-electrode system lacks reference electrodes but the three-electrode system has a reference electrode, and this reference electrode is mainly responsible for current amplification of the developed biosensor [17]. The designed aptasensor is based on aptamer, and aptamer is one of the bioreceptors utilized due to its various advantages, such as simple synthesis, low cost, and stability, which can also be applicable for real samples such as short DNA or RNA oligonucleotides chosen in vitro to bind a particular target that have similar binding affinities to antibodies but better chemical and biological stabilities. Hence, through the development of a dengue aptasensor, this research aims to examine the variance in the currents of both electrode setups. This study not only compares the currents but also compares the fabrication methods of both paper electrode setups, and along with this, their key features, and lastly, the justification for using the three-electrode system are also depicted.

## 2. Materials and Methods

### 2.1. Chemicals, Reagents, and Apparatus

Methylene blue was purchased from Sigma Aldrich, India, and all other chemicals were of AR grade. For the creation of paper electrode setups, A-4-size sheets in addition to silver conductive paste and black carbon conductive ink were acquired from Snab Graphix Pvt. Ltd. (Bangalore, India) NaCl, KCl, Na_2_HPO_4_, and KH_2_PO_4_ were acquired from LOBA to prepare PBS. The aptamer and antigen were prepared in PBS (pH 7.4, 100 M). The DENV-aptamer, a 34-base oligonucleotide, was purchased from MTOR life science Pvt. Ltd. and was complementary to the polyvalent DENV-antigen (Prospec-Tany TechnoGene Ltd. Israel).

Aptamer sequence: GCACCGGGCAGGACGTCCGGGGTCCTCGGGGGGC [18]. Metrohm Dropsens (Stat-I 400s) were used for electrochemical experiments involving cyclic voltammetry (CV). Screen-printing frames were designed and purchased from a local project shop.

### 2.2. Fabrication and Features of Different Paper Electrode Setups

**Scheme 1:**
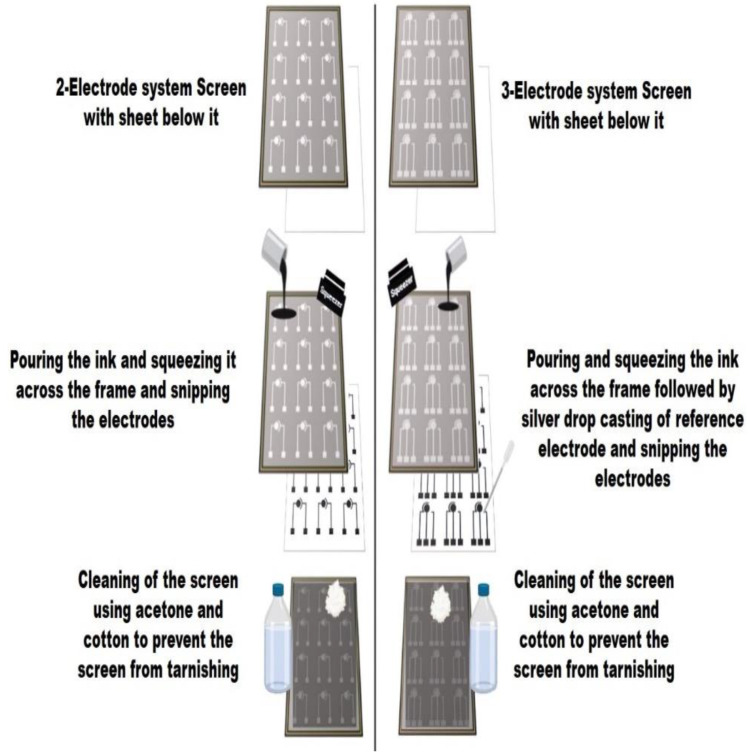
A comparative schematic representation of fabrication of two- and three-electrode systems.

In this study, two setups—the (a) two-electrode system and (a,b) three-electrode system—were constructed. Both of these systems were created using the screen-printing technique. In this technique, hand printing was performed using a silk screen with a laser-cut patterned solid skin that was adhered to it and that had predetermined dimensions for a three-electrode arrangement. Carbon conductive ink (mainly composed of graphene, as it is a good conductor [19,20] was squeezed onto cellulose sheets using a squeezer via the designated overhead screen’s open areas. The silk screen was used as a stencil for the electrode preparation once the electrode’s dimensions were set and framed on it. Three electrodes—a counter electrode (CE), a working electrode (WE), and a reference electrode (RE) drop cast with Ag/AgCl—made up the printed electrodes. As a result of this, the a three-electrode system was created, whereas the production of a two-electrode system involved all the same stages except for the fabrication of the reference electrode (Scheme 1 & Table 1).

### 2.3. Construction of Dengue Aptasensor to Check the Current Range of Both Electrode Setups

Firstly, silver/zinc nanocomposites were chemically synthesized, which were then deposited to the working electrode of both electrode setups (two-electrode and three-electrode system) and dried overnight, and then the aptamer was immobilized on both working electrodes the following day. Finally, dengue antigen was drop-casted on both these electrodes previously containing silver/zinc nanocomposites, and at last, methylene blue was deposited. CV was then performed and recorded for both two- and three-electrode systems. The dengue virus was successfully detected using the electrode setups, showing different current ranges. In the results section, these current variation comparisons are discussed in more detail.

## 3. Results

### 3.1. Comparative Study of Current Amplification Based on Three- and Two-Electrode Setup, and Summarizing Their Differences in the Currents-

#### 3.1.1. Current Comparison of Different Stages of Aptasensor

**Figure 1:**
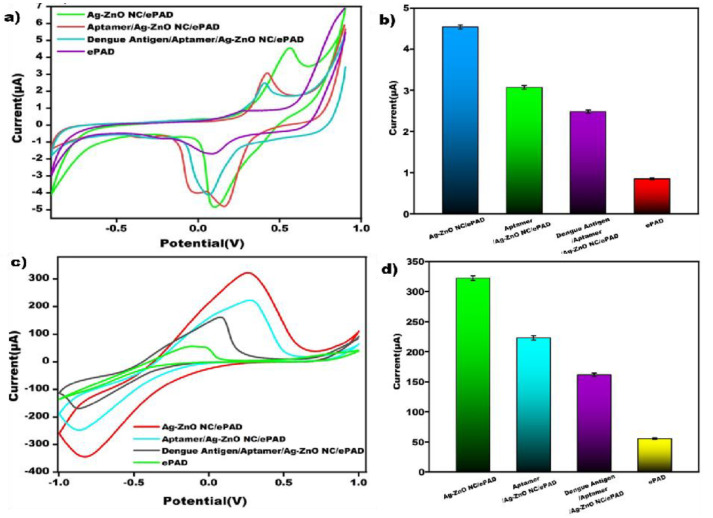
(**a**) CV of different stages of aptasensor showing different current ranges based on the two-electrode system. (**b**) Bar graph represents the different current ranges based on the two-electrode system. (**c**) CV of different stages of aptasensor showing different current ranges based on three-electrode system. (**d**) Bar graph represents the different current ranges based on the three-electrode system.

For comparative study, first, we compared the currents of different stages of developing a dengue aptasensor. The four stages are: nanoparticles, aptamer, dengue antigen, and bare, which display different current responses based on two- and three-electrode systems. All these current range comparisons were confirmed by cyclic voltammetry (CV). Figure 1a,b show the differential current response at different stages of the electrode. In CV, bare electrodes displayed a lower peak current response, which is a consequence of slower electron transfer kinetics (Table 2). Due to the fast electron transfer kinetics offered by the silver/zinc nanocomposite, there was a large improvement in current responsiveness following its deposition onto the working surface. The non-conductive nature of the aptamer after immobilization onto the working surface significantly reduced the current. The well-known MB principle led to a further reduction in the current response after the introduction of an antigen. Intercalation of MB between bases drastically reduced the current response. This principle of stages is the same in both electrode setups.

#### 3.1.2. Current Comparison of Different Concentrations of Dengue Antigen

**Figure 2:**
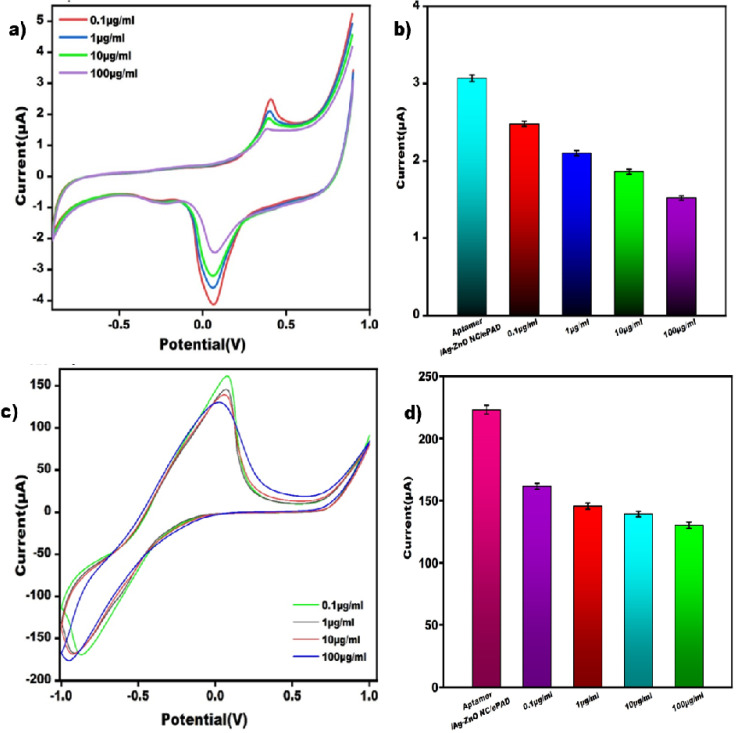
(**a**) CV of different concentrations of dengue antigen showing different current ranges based on the two-electrode system. (**b**) Bar graph of different concentrations based on the two-electrode system **.** (**c**) CV of different concentrations of dengue antigen showing different current ranges based on the three-electrode system. (**d**) Bar graph of different concentrations based on the three -electrode system.

The currents of different concentrations of the developed dengue aptasensor were compared. It involved four different concentrations of dengue antigen, i.e., 0.1, 1, 1.0, and 100 μg/mL, which showed different current ranges based on two- and three-electrode systems. This current comparison of the developed aptasensor was validated by cyclic voltammetry (CV) (Table 3). A variety of concentrations, including 0.1, 1, 10, and 100 g/mL, were used in Figure 2a,b to facilitate aptamer hybridization. The findings showed that the antigen exhibits aptamer hybridization and that variable current responses were seen at various concentrations, confirming the constructed sensor’s quantitative functionality. The outcomes are consistent with previously reported sensors. Since more insulating layers of the biological recognition element slow down the electron transfer, the current response decreased when antigen concentrations were increased. This principle of comparing the currents of different concentrations was the same in both electrode setups.

Based on the above results, it is very clear why the three-electrode system is best compared to the two-electrode system in terms of high electron transfer, high current amplification, and its unique electrochemical principles as well.

### 3.2. Justification for Using Three-Electrode System over Two-Electrode System

**Figure 3:**
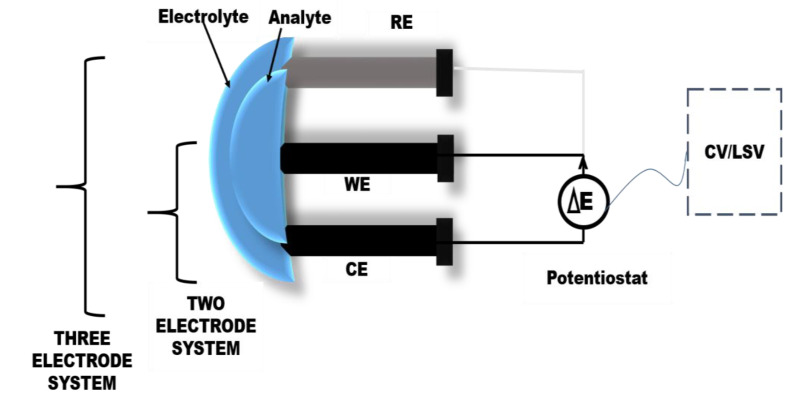
Working principle of two-electrode and three-electrode electrochemical setups.

In the construction of a two-electrode system, only the counter and working electrodes are used. To begin the dengue virus detection mechanism, we first established the sensor by depositing nanoparticles on the circular region of the working electrode, then immobilized aptamer on the nanoparticles, and finally deposited dengue antigen followed by methylene blue (MB), which acts as an intercalating agent. Both electrodes were immersed in MB solution, and the current response was measured and confirmed using several electrochemical parameters such as CV/LSV. The response was measured upon the imposition of **ΔE** potential between the two electrodes [21]. The sensing protocol was the same in both electrode systems except that the reference electrodes were also dipped in MB along with counter and working electrodes, which significantly helps in enhancing the current range of the three-electrode-based biosensor [22] (Figure 3).

**The two-electrode system** is made up of a working electrode, where the chemistry of interest occurs, and a counter electrode, which serves as the other half of the cell. The working and counter electrode potentials are measured, and the resulting current is measured in the working or counter electrode lead. In the two-electrode setup, the counter electrode serves two purposes. It completes the circuit, enabling charge to flow through the cell, and it also maintains a constant inter-facial potential irrespective of current. It is extremely difficult to maintain a constant counter electrode potential in a two-electrode system when current flows. **The three-electrode system,** on the other hand, addresses many of the shortcomings of the two-electrode configuration. A working electrode, a counter electrode, and a reference electrode comprise the three-electrode system. The role of the reference electrode is to serve as a reference in measuring and attempting to control the working electrode potential while no current is passed through it. At low current density, the reference electrode has a constant electrochemical potential. Furthermore, because the reference electrode passes negligible current, the iR drop between the reference and working electrodes is frequently very small. Thus, with the three-electrode system, the reference potential is much more stable, and there is compensation for the iR drop across the solution. This results in greater control over the working electrode potential. The counter electrode’s function in the three-electrode configuration is to pass all of the current required to balance the current observed at the working electrode. To accomplish this task, the counter electrode frequently swings to extreme potentials.

## 4. Conclusions and Future Perspective

In this study, both paper electrode setups were successfully fabricated with the help of the screen-printing method, and we utilized these electrodes for the construction of a dengue aptasensor by employing silver/zinc nanocomposites. Both electrode setups were successfully capable in the determination of dengue virus, but the three-electrode system showed high current amplification as compared to the two-electrode system. Therefore, we can predict that in the future, two-electrode systems will be completely replaced by the three-electrode system. This report gives the scientific community deeper insight into the electrode systems in terms of current amplification, fabrication methods, and their comparative features. In the future, such studies may be extremely beneficial to new researchers for obtaining good knowledge of the paper-based electrode setup, types of electrode setups, manufacturing technique of electrodes, different mechanisms of electrodes, and comparison of the two- and three-electrode systems. The present results show that the three-electrode arrangement is superior to the two-electrode setup. As a result, we will continue our research on the dengue aptasensor based on the three-electrode system only (and dropping the idea of the two-electrode system) by optimizing it on different parameters, trying to validate their results on human serum, and also modifying these three-electrode systems by using a paper-folding technique called origami, which may lead to the development of a novel report, i.e., origami-based dengue aptasensor.

## 5. Discussion

No such reports are available on the comparison of paper electrode setups in terms of current enhancement. Two previous studies are compared in Table 4, which clearly reveals the current amplification using two- and three-electrode systems. The present work has shown a higher current amplification system as compared to previous studies.

## Figures and Tables

**Table 1:** Comparative features of different paper electrode setups.

S.No	Involvements	Two-Electrode System (a)	Three-Electrode System (b)
**1.**	**Total electrodes**	Two electrodes	Three electrodes
**2.**	**Name of electrodes**	-Working electrode (WE) -Counter electrode (CE)	-Working electrode (WE) -Counter electrode (CE) -Reference electrode (RE)
**3.**	**Ink/paste**	Black carbon conductive ink	-Black carbon conductive -Silver paste casting for reference electrode
**4.**	**Substrate**	Paper (A-4-size sheets)	Paper (A-4-size sheets)
**5.**	**Fabrication method**	Screen-printing method	Screen-printing method
**6.**	**Incubation time**	Overnight	Overnight
**7.**	**Electrochemical** **measurement for dengue detection**	Cyclic voltammetry technique	Cyclic voltammetry technique
**8.**	**Current range**	Low	Very high
**9.**	**Expected price per electrode (INR)**	2 rupees (approx.)	5 rupees (approx.)
**10.**	**Preparation time**	3 min	5 min
**11.**	**Usability**	Prepare, use, and throw	Prepare, use, and throw

**Table 2:** Summarized the comparison of the different current ranges of the aptasensor stages based on different paper electrode setups.

S.No	Different Stages	Current (Two-Electrode Setup)	Current (Three-Electrode Setup)
**1.**	**Nanocomposites**	4.54 µA	322.21 µA
**2.**	**Aptamer**	3.07 µA	223.04 µA
**3.**	**Antigen**	2.48 µA	161.44 µA
**4.**	**Bare**	0.85 µA	55.53 µA

**Table 3:** Summarized the comparison of the different current ranges of the different concentrations of dengue antigen based on different paper electrode setups.

**S.No**	**Dengue Antigen Concentrations**	**Current (Two-Electrode Setup)**	**Current (Three-Electrode Setup)**
**1.**	**0.1 µg/mL**	2.48 µA	161.44 µA
**2.**	**1 µg/mL**	2.10 µA	145.57 µA
**3.**	**10 µg/mL**	1.86 µA	139.20 µA
**4.**	**100 µg/mL**	1.52 µA	130.19 µA

**Table 4:** Comparison of existing reports with the present work.

Biosensor	Electrode System	Electrodes involved	Current Range (Approx.)	References
Electrochemical biosensor (zika virus)	Two-electrode system	Counter and working electrodes	2 µA	[20]
Electrochemical biosensor (yellow fever)	Three-electrode system	Counter, working, and reference electrodes	180 µA	[15]
Electrochemical biosensor (dengue virus)	Three-electrode system	Counter, working, and reference electrodes	322.21 µA	This work

## Data Availability

Data will be made available on request.
